# Microbial Allies from the Cold: Antarctic Fungal Endophytes Improve Maize Performance in Water-Limited Fields

**DOI:** 10.3390/plants14142118

**Published:** 2025-07-09

**Authors:** Yessica San Miguel, Rómulo Santelices-Moya, Antonio M. Cabrera-Ariza, Patricio Ramos

**Affiliations:** 1 Plant Microorganism Interaction Laboratory, Instituto de Ciencias Biológicas, Universidad de Talca, Talca 3460787, Chile; yessica.sanmiguel@utalca.cl; 2 Centro del Secano, Facultad de Ciencias Agronómicas y Forestales, Universidad Católica del Maule, Talca 3466706, Chile; rsanteli@ucm.cl; 3 Centro de Investigación de Estudios Avanzados del Maule, Vicerrectoría de Investigación y Postgrado, Universidad Católica del Maule, Talca 3466706, Chile

**Keywords:** climate change, water scarcity, Antarctic fungal endophytes, plant resilience

## Abstract

Climate change has intensified drought stress, threatening global food security by affecting sensitive crops like maize (*Zea mays*). This study evaluated the potential of Antarctic fungal endophytes (*Penicillium chrysogenum* and *P. brevicompactum*) to enhance maize drought tolerance under field conditions with different irrigation regimes. Drought stress reduced soil moisture to 59% of field capacity. UAV-based multispectral imagery monitored plant physiological status using vegetation indices (NDVI, NDRE, SIPI, GNDVI). Inoculated plants showed up to two-fold higher index values under drought, indicating improved stress resilience. Physiological analysis revealed increased photochemical efficiency (0.775), higher chlorophyll and carotenoid contents (45.54 mg/mL), and nearly 80% lower lipid peroxidation in inoculated plants. Lower proline accumulation suggested better water status and reduced osmotic stress. Secondary metabolites such as phenolics, flavonoids, and anthocyanins were elevated, particularly under well-watered conditions. Antioxidant enzyme activity shifted: SOD, CAT, and APX were suppressed, while POD activity increased, indicating reprogrammed oxidative stress responses. Yield components, including cob weight and length, improved significantly with inoculation under drought. These findings demonstrate the potential of Antarctic endophytes to enhance drought resilience in maize and underscore the value of integrating microbial biotechnology with UAV-based remote sensing for sustainable crop management under climate-induced water scarcity.

## 1. Introduction

Climate change is increasingly threatening food security, with global warming posing major challenges to agricultural production worldwide. Extended droughts and high temperatures are reducing crop yields [1], especially in developing countries where drought stress severely limits agricultural output [2,3]. Drought occurs when soil moisture falls below critical levels, reducing turgor pressure, which negatively affects water potential and disrupts plant growth and physiological functions [4,5].

Under water stress, plants typically exhibit reduced growth, lower leaf water content, decreased turgor pressure, and diminished transpiration rates [6]. Prolonged drought affects vital cellular processes, including protein synthesis, nutrient uptake, and photosynthesis [6,7]. In response, plants accumulate osmolytes to maintain turgor pressure, relative water content, and stomatal conductance, stabilize membranes, regulate reactive oxygen species (ROS), and support overall growth [7,8].

Maize (*Zea mays* L.) is the third most consumed cereal globally, with annual production exceeding one trillion tons and demand projected to double by 2050 [9]. However, maize is highly vulnerable to drought, particularly during early growth, vegetative, and reproductive stages [10].

In nature, plants coexist with various microorganisms that may benefit, harm, or have neutral effects on growth. Fungal colonization of roots often enhances nutrient uptake and stress tolerance [11]. In agriculture, these beneficial microbes can be leveraged as plant growth-promoting agents or biocontrol organisms [11].

Antarctica, one of the harshest environments—characterized by extreme cold, limited water, high UV-B radiation, and saline soils [12]—hosts only two vascular plants: *Deschampsia antarctica* and *Colobanthus quitensis* [13]. Endophytic fungi isolated from these plants have demonstrated the ability to enhance stress tolerance in other host plants [14,15,16,17,18,19].

Integrating UAV-based hyperspectral and multispectral imaging provides advanced tools for monitoring crop health and stress responses under varying environmental conditions. Vegetation indices (VIs), such as the normalized difference vegetation index (NDVI), are effective in detecting physiological changes due to drought or chemical stress, helping reduce labor-intensive fieldwork [20]. Dynamic indices like the green NDVI (GNDVI) further support real-time monitoring by correlating with plant height and water potential, aiding precision irrigation [21].

Hyperspectral imaging offers additional precision. For instance, UAV hyperspectral data to monitor maize canopy chlorophyll density (CCD) under lodging stress, successfully predicting lodging severity (R^2^ = 0.63) [22]. Similarly, a study showed that removing background elements from UAV hyperspectral images significantly improved SPAD value estimation in maize, enhancing accuracy and enabling better detection of nutrient deficiencies and other stress indicators [23].

These innovations are especially valuable for drought-sensitive crops like maize. UAV-derived indices such as the crop water stress index (CWSI) and enhanced vegetation index (EVI2) allow detailed assessments at canopy and plant levels [24], facilitating genotype evaluations and selection of drought-tolerant varieties. Additionally, UAV-based VIs assist in estimating yield and protein content throughout growth stages, improving nitrogen management and planting density decisions [25].

In this study, we demonstrate that two Antarctic fungal endophytes, *Penicillium chrysogenum* and *P. brevicompactum*, isolated from the roots of Antarctic vascular plants, enhanced drought tolerance in field-grown maize. Biochemical and physiological analyses indicate that symbiosis with these endophytes helps plants cope with water stress, minimizing yield losses. These findings were further supported by vegetation indices and UAV-based multispectral imaging, which validated improved plant performance under drought conditions.

## 2. Results

### 2.1. Soil Water Level Estimation

The experiments were carried out under field conditions, where soil moisture and water availability are not so easy to control, and the water content in the soil was determined on the soil immediately under each group of plants treated. The soil water content was analyzed in the soil immediately under the plants, considering 60 cm depth. Table 1 shows the field capacity (FC) expressed as the percentage of soil water content compared to well-watered soil (100%). Deficient irrigation is 59% of the FC compared to the control water treatment.

### 2.2. Spectral Images Analysis

**Figure 1:**
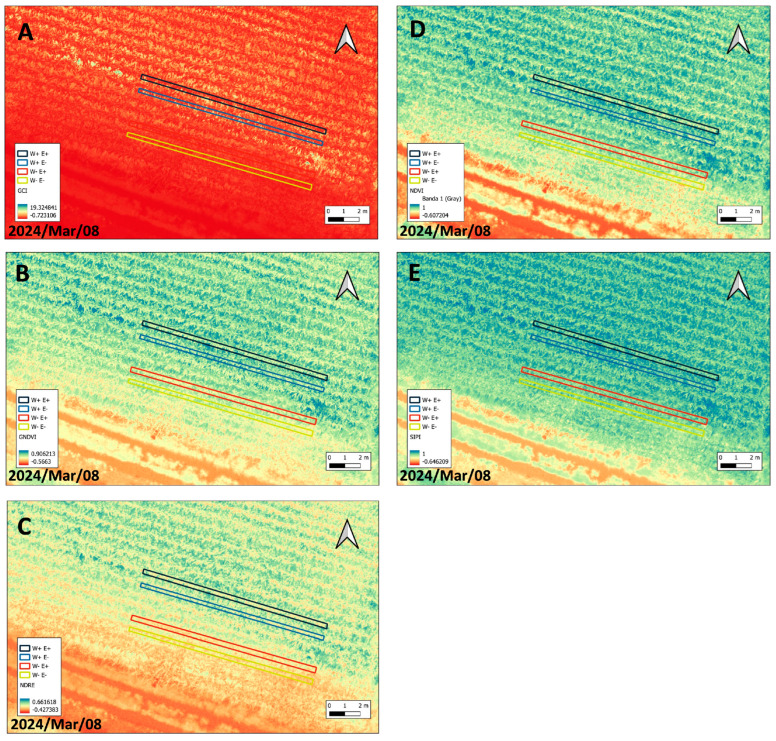
UAV-acquired images of 50 maize plants per treatment under deficient and full irrigation regimens. Vegetation indices assessed included the (**A**) Green Chlorophyll Index (GCI), (**B**) Green Normalized Difference Vegetation Index (GNDVI), (**C**) Normalized Difference Red Edge (NDRE), (**D**) Normalized Difference Vegetation Index (NDVI), and (**E**) Structure Insensitive Pigment Index (SIPI). Colored selections indicate groups of well-watered (W+) and deficient-irrigated plants (W−), either inoculated (E+) or non-inoculated (E−).

An analysis of UAV-acquired images was conducted to evaluate maize plants subjected to deficient and full irrigation regimens. Various vegetation indices were assessed, including the Green Chlorophyll Index (GCI), Green Normalized Difference Vegetation Index (GNDVI), Normalized Difference Red Edge (NDRE), Normalized Difference Vegetation Index (NDVI), and Structure Insensitive Pigment Index (SIPI) (Figure 1). Figure 1 shows the images obtained from the treatments, highlighting qualitative differences in the intensity of foliar damage. These differences were most severe in non-inoculated plants under water deficit conditions (W−E−) compared to other treatments.

**Figure 2:**
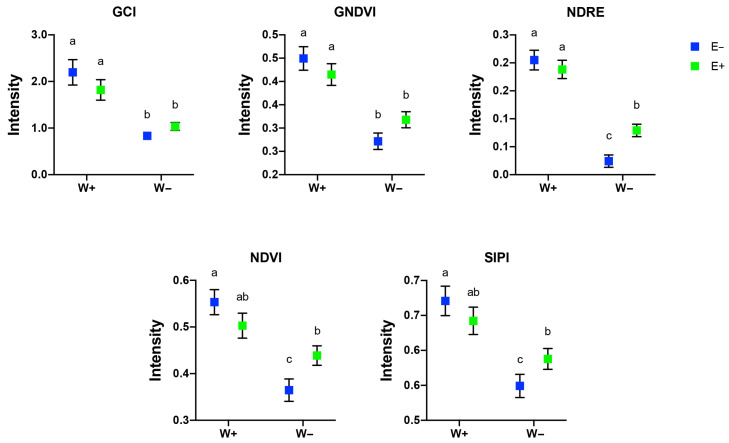
Analysis of pixel information quantifying signal intensity based on heat bar data for the Green Chlorophyll Index (GCI), Green Normalized Difference Vegetation Index (GNDVI), Normalized Difference Red Edge (NDRE), Normalized Difference Vegetation Index (NDVI), and Structure Insensitive Pigment Index (SIPI). Different letters indicate significant differences (*p* < 0.05; two-way ANOVA). Bars represent means ± SE from three independent experiments.

To obtain more quantitative results of the vegetation indexes, an image analysis was performed using pixel information, quantifying the signal intensity based on the heat bar information of each index. All the indexes evaluated did not show significant differences between inoculated and non-inoculated well-watered plants (W+E+ and W+E−, respectively). In plants exposed to a water deprivation regimen, GCI and GNDVI showed no differences between non-inoculated and inoculated plants (Figure 2). Interestingly, the analysis of NDRE, NDVI, and SIPI showed that fungal inoculation induces a significant improvement of these indexes in plants under water-deficient conditions (W−E+) (Figure 2).

### 2.3. Physiological and Biochemical Parameters of Plant Response to Drought and Fungal Symbiosis

To determine whether maize plants experienced stress due to water deprivation in the field, we conducted several assessments, including measurements of photochemical efficiency, chlorophyll and carotenoid content, free proline concentration, and membrane damage. Fv/Fm, an indicator of the maximum quantum efficiency of photosystem II (PSII), is commonly used to assess the photosynthetic performance and stress status of plants. All plants grew similarly within each treatment group and were included in the analysis. Physiological and biochemical assays were performed at the end of the 90-day treatment period. When plants experience environmental stress, this value typically drops below 0.80. The Fv/Fm values were evaluated at the harvest. The group of non-inoculated plants under deficient water regimen (W−E−) displays a significant reduction in the photochemical efficiency compared to control well-watered and non-inoculated and inoculated plants (W+E− and W+E+) (Table 2). Inoculated plants under water deficit showed an increased photochemical efficiency compared to the non-inoculated (W−E+ and W−E−).

Concerning plant pigments, the chlorophyll a and chlorophyll b content were higher in inoculated plants experiencing water deficit (W−E+) compared to non-inoculated plants under the same conditions (W−E−). Inoculated well-watered plants display increased contents of chlorophylls compared to non-inoculated plants under the same water regimen (W+E+ and W+E−, respectively). Additionally, significant differences in carotenoids were observed among inoculated well-watered plants (W+E+), uninoculated well-watered plants (W+E−), and uninoculated plants subjected to drought (W−E−) (Table 3).

**Figure 3:**
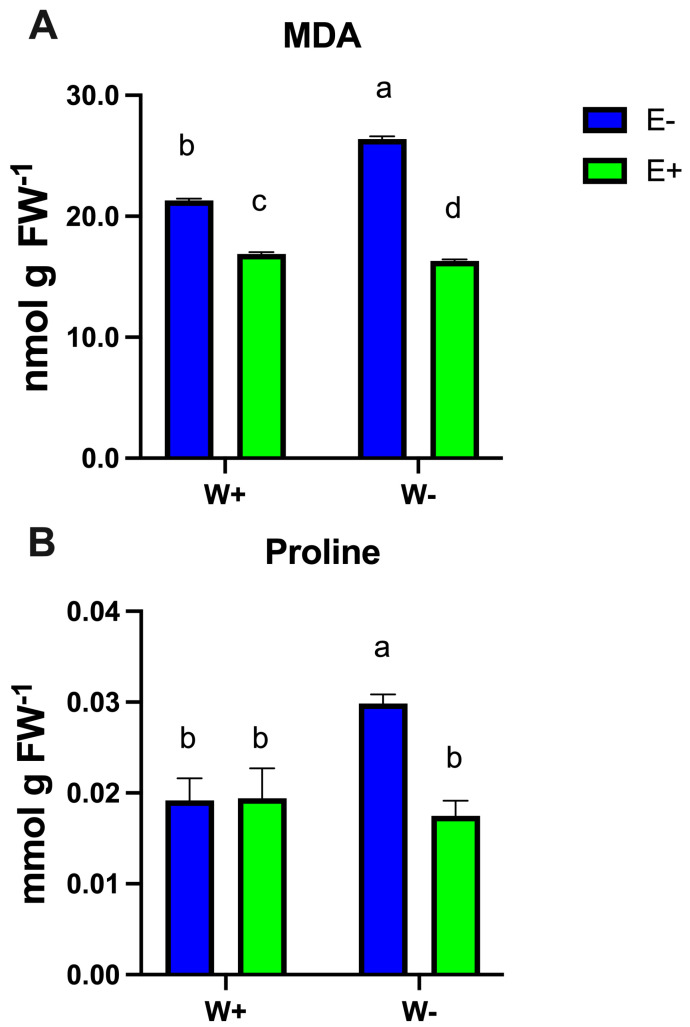
Concentration of MDA indicative of lipid peroxidation (**A**) and proline (**B**) in maize plants inoculated (E+) or non-inoculated (E−) exposed to contrasting water availability conditions (drought: W−, well-watered: W+). Bars represent means ± S.E. Different letters indicate significant differences between means (Tukey’s HSD; *p* < 0.05).

To assess whether fungal symbiosis influences factors related to abiotic stress tolerance, we evaluated markers of cell damage caused by oxidative lipid degradation (Figure 3A). Lipid peroxidation—a well-established indicator of cell damage—was measured using the thiobarbituric acid reactive substance (TBARS) assay to quantify malondialdehyde (MDA) concentrations [26]. MDA levels increased in non-inoculated plants under water-deficit conditions; however, this effect was effectively mitigated by fungal inoculation under both well-watered and drought conditions (Figure 3A).

Regarding the biochemical response, the amino acid proline has been a central focus in studies investigating how endophytes influence plant stress responses due to its multiple roles in alleviating stress in plants [27,28,29]. Our analysis revealed that inoculated plants (E+) under water-deficient conditions (W−) had lower proline levels compared to non-inoculated plants (E−), suggesting that fungal inoculation may reduce the need for proline accumulation as a protective response to drought stress (Figure 3B).

### 2.4. Phenolics Antioxidant Compounds

**Figure 4:**
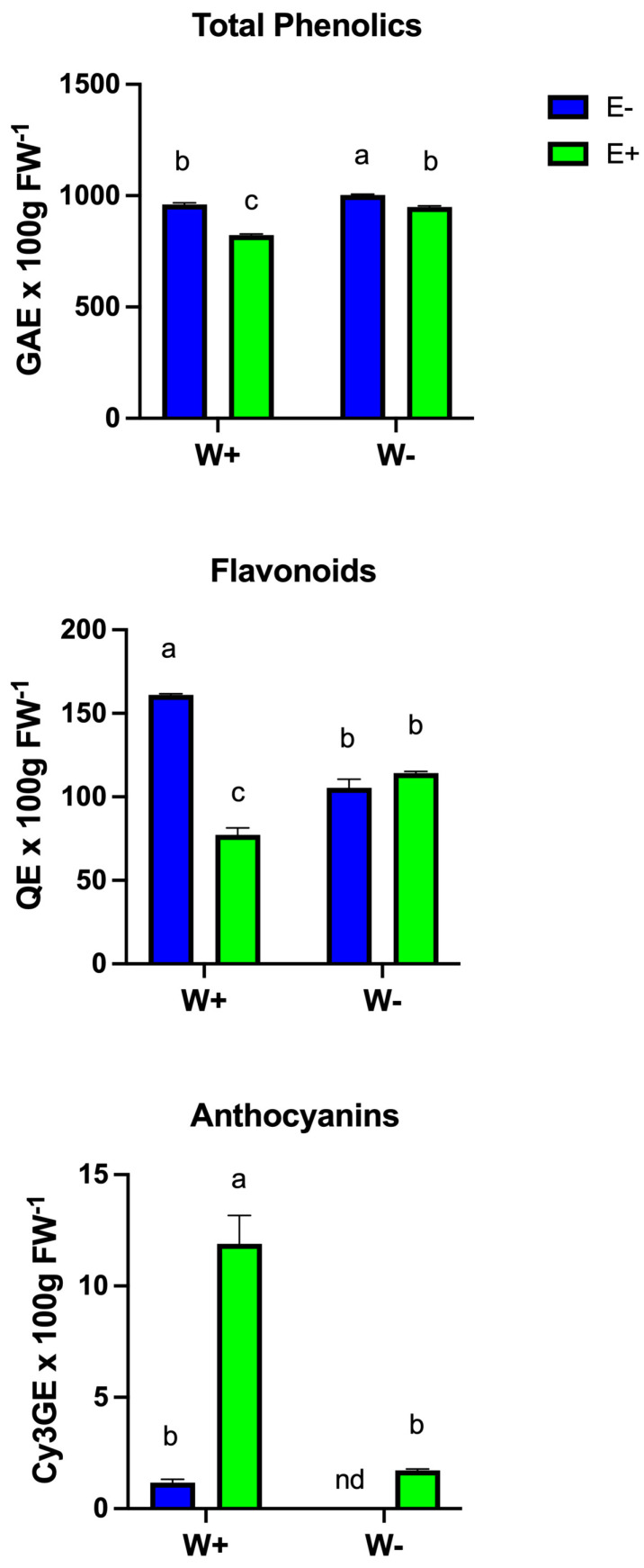
Total phenolics, flavonoids, and anthocyanins were evaluated in the leaves of plants under each treatment of well-watered (W+) and deficient-irrigated plants (W−), either inoculated (E+) or non-inoculated (E−). Bars represent means ± SE from three independent experiments. Different letters indicate significant differences between means (Tukey’s HSD; *p* < 0.05).

Total phenolics, flavonoids, and anthocyanins were evaluated in the leaves of plants under each treatment (Figure 4). Total phenolic content was higher in water-stressed plants compared to well-watered ones. Interestingly, the presence of fungal endophytes reduced phenolic content under both water regimes relative to non-inoculated plants.

In terms of total flavonoids, their content significantly decreased in plants exposed to water deficit compared to well-watered conditions. Under well-watered (W+) conditions, non-inoculated plants exhibited significantly higher flavonoid content than inoculated ones. However, under water-deficient (W−) conditions, total flavonoid levels in inoculated plants were comparable to those in non-inoculated plants. This suggests that inoculation helps sustain flavonoid levels during water stress, mitigating the decline typically observed in non-inoculated plants. Therefore, inoculation appears more beneficial under water-deficit conditions, where it contributes to the maintenance of flavonoid content, while under well-watered conditions, its effect on flavonoid production is neutral or slightly negative. Inoculated plants exhibited significantly higher anthocyanin content than non-inoculated plants, suggesting that inoculation promotes anthocyanin accumulation under well-watered conditions (W+E+ and W+E−). Non-inoculated plants showed low anthocyanin levels, indicating a minimal response in the absence of inoculation under well-watered treatment (W+E−). Under water-deficit conditions (W−E−), anthocyanin content in non-inoculated plants was negligible (not detected, “nd”). Inoculated plants under water deficit (W−E+) had low anthocyanin levels, but these were significantly higher than those of non-inoculated plants and comparable to levels observed in non-inoculated plants under well-watered conditions (W+E−). Notably, inoculation dramatically increased anthocyanin content under well-watered conditions (W+E+) and maintained a modest increase under water-deficit conditions (W−E+) (Figure 4).

### 2.5. Enzymatic Antioxidant Activity

**Figure 5:**
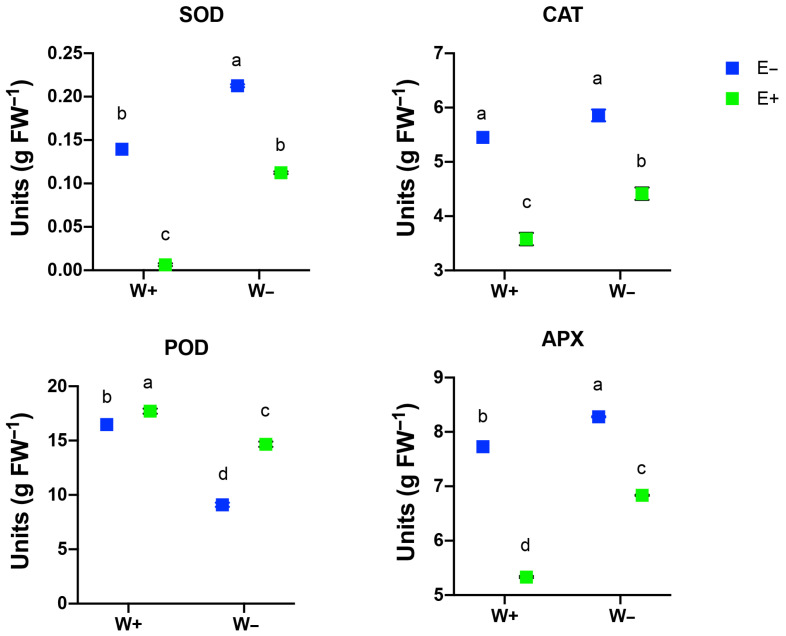
Effect of water deficit treatments (W+ and W−) on the activities of the antioxidant enzymes (CAT, POD, SOD, and APX) in leaves of maize plants inoculated (E+) or non-inoculated (E−) with the root fungal endophytes. Bars represent means ± S.E. Different letters indicate significant differences between means (Tukey’s HSD; *p* < 0.05).

Enzymatic antioxidant activity was also evaluated in the leaves under different treatments (Figure 5). The results showed that the activities of SOD, CAT, and APX followed a similar pattern, increasing their activity under water deficit conditions but strongly inhibited by fungal inoculation. As for POD enzymatic activity, it was reduced in plants under water deficit conditions compared to well-watered plants. However, in both water treatments, its activity significantly increased in the presence of fungi (Figure 5).

### 2.6. Cob Analysis

**Figure 6:**
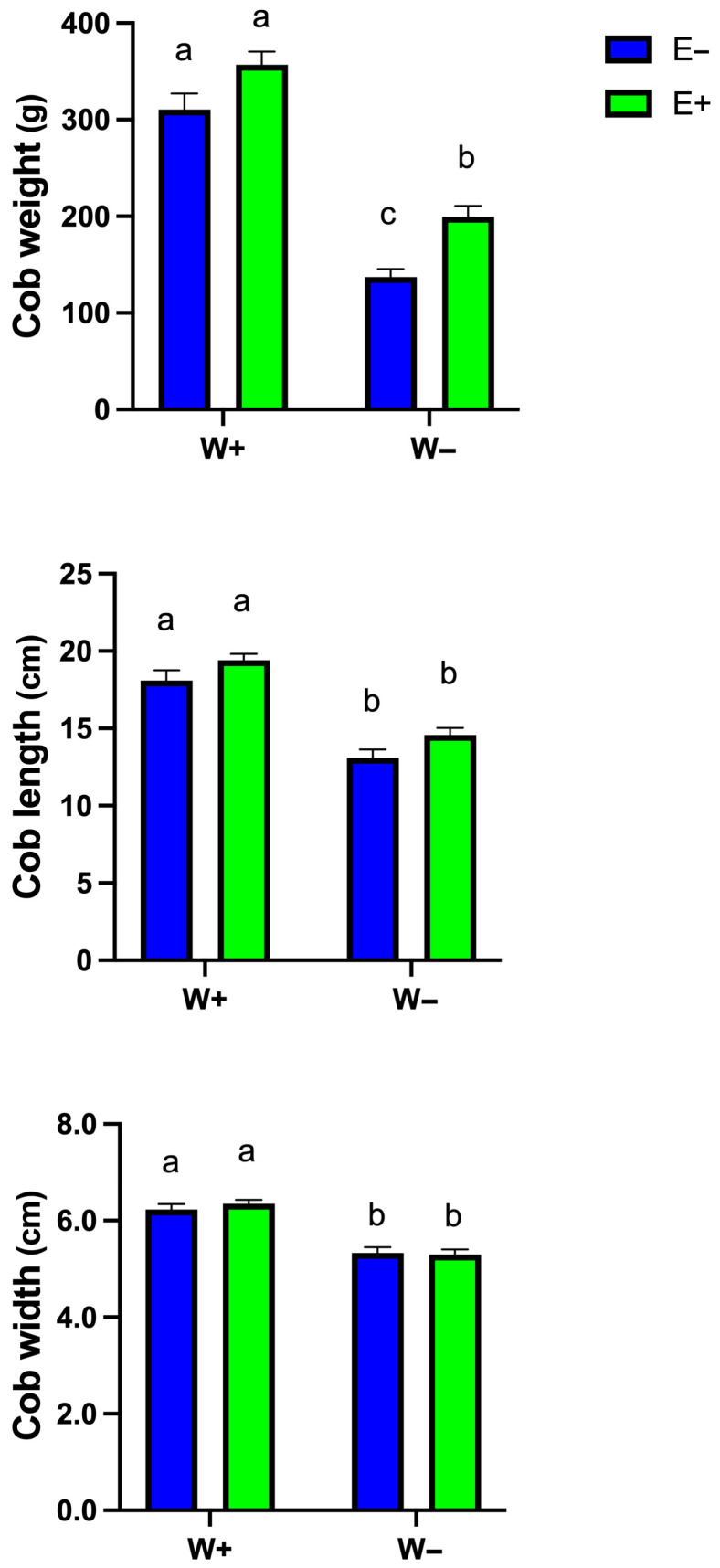
Effects of water availability (W+ and W−) and inoculation (E− and E+) on cob weight, length, and width. Bars represent means ± S.E. Different letters indicate significant differences between means (Tukey’s HSD; *p* < 0.05).

Figure 6 shows the effects of water availability (W+ = well-watered, W− = water-deficient) and inoculation (E− = non-inoculated, E+ = inoculated) on cob weight, length, and width. In the case of weight, well-watered and inoculated plants (W+E+) show slightly higher weights than non-inoculated ones (W+E−), although the difference is not statistically significant. This indicates that inoculation might provide minor benefits under sufficient water. Cob of water-deficient and inoculated plants (W−E+) show significantly higher weights than non-inoculated ones (W−E−). This demonstrates that inoculation alleviates drought stress, possibly through enhanced water and nutrient uptake, allowing the plant to maintain cob production.

The length of cobs shows that both W+E+ and W+E− groups have similar lengths, indicating that inoculation does not significantly affect cob growth when water is not a limiting factor.

In water-deficient plants, cob length is reduced for both groups (W−E+ and W−E−). However, in inoculated plants (E+), the cobs maintain longer lengths compared to non-inoculated ones (E−), highlighting the role of inoculation in mitigating drought-induced reductions in cob development.

Regarding the width, this character remains uniform in both E+ and E−, showing no significant effect of inoculation under optimal conditions (W+). However, under water-deficient treatment, both inoculated and non-inoculated groups exhibit reduced cob width compared to W+. However, there is no statistical difference between inoculated and non-inoculated cobs under deficient irrigation, suggesting that inoculation primarily supports weight and length rather than cob width during stress.

## 3. Discussion

Our findings demonstrate that inoculation with fungal endophytes significantly enhances the physiological performance of maize under drought stress, as indicated by elevated values of NDRE, NDVI, and SIPI. These results are consistent with previous studies that highlight the utility of spectral indices in assessing plant health and stress responses [30,31]. The observed increases in NDRE and NDVI among inoculated, drought-stressed plants suggest enhanced chlorophyll content and photosynthetic efficiency, supporting the hypothesis that fungal endophytes help mitigate drought-related stress. The utility of UAV-based spectral analysis for monitoring maize physiology is well documented [32]. UAV-derived vegetation indices—particularly those incorporating red-edge and near-infrared (NIR) bands—are widely used to estimate key canopy traits such as chlorophyll content and biomass accumulation [30,31]. Our results reinforce these findings, demonstrating that UAV multispectral imagery effectively captures physiological differences resulting from fungal endophyte inoculation under water-limited conditions. Similarly, previous research has employed UAV-based multispectral imaging to estimate leaf area index (LAI) under varying irrigation and nutrient regimes, highlighting its potential for quantifying crop responses. Our findings complement this work by showing that UAV-derived indices can also detect physiological improvements associated with microbial symbiosis during drought stress [33].

Interestingly, no significant differences were observed between inoculated and uninoculated plants under well-watered conditions across all spectral indices. This suggests that under non-stress conditions, fungal inoculation does not confer additional physiological benefits detectable via spectral analysis. These findings align with previous studies indicating that plant–microbe interactions often exhibit more pronounced effects under stress conditions [30,32].

In contrast, the observed improvements in NDRE, NDVI, and SIPI in drought-stressed, inoculated plants suggest that endophytes contribute to maintaining chlorophyll integrity and mitigating oxidative stress, both critical for sustaining photosynthesis under adverse conditions. Similar trends have been reported in biomass estimation studies, where vegetation indices derived from red-edge and near-infrared (NIR) bands play a central role in predicting crop performance under stress [30]. Elevated SIPI values further support the protective role of endophytes, as SIPI reflects the carotenoid-to-chlorophyll ratio, an established indicator of oxidative stress tolerance [31]. Using UAV-derived data for plant monitoring offers a non-destructive and scalable approach for assessing physiological responses to both biotic and abiotic stressors. Our findings underscore the potential of integrating remote sensing technologies with microbial inoculation strategies to enhance crop resilience under water-limited conditions. Future research should explore the long-term effects of these interactions across diverse maize genotypes and environmental contexts, as well as the application of machine learning techniques for automated stress detection [30,31].

In addition, drought stress resulted in elevated malondialdehyde (MDA) levels—an indicator of lipid peroxidation and oxidative damage—while fungal endophyte inoculation significantly mitigated this response (Figure 3A), suggesting enhanced protection against oxidative stress. These findings align with previous studies reporting reduced MDA accumulation following fungal inoculation, including a 25.06% reduction achieved using a consortium of three endophytes [34], and approximately 30% reduction observed in blueberries inoculated with Antarctic fungi [18]. Plants respond to drought by producing osmolytes to adjust water potential. Proline is a key osmolyte that maintains turgor pressure and enables stomatal function under water stress [35]. It also contributes to non-enzymatic antioxidant defenses [36,37]. Elevated proline accumulation under drought has been documented in endophyte-colonized plants, likely because of microbial-induced metabolic reprogramming [17,38]. For instance, drought-stressed rice inoculated with *T. harzianum* exhibited higher levels of proline and ROS-scavenging enzymes [39]. However, in our study, increased proline levels were only observed in uninoculated, drought-stressed plants, with no significant change in inoculated counterparts (Figure 3B). These results are consistent with prior findings, such as those by Kavroulakis et al. (2018) [35], where Fusarium-inoculated plants did not accumulate proline under stress. Similarly, Rodríguez et al. (2008) [40] reported decreased proline levels in tomato plants colonized by *F. culmorum*, suggesting that endophytes may mitigate the need for osmotic adjustment via proline.

Mycorrhizal symbiosis may also explain this response, as fungal hyphae facilitate water uptake and maintain soil–plant water continuity, reducing the need for osmotic compensation [41,42]. Abiotic stress elevates reactive oxygen species (ROS), particularly H_2_O_2_, which causes oxidative damage and lipid peroxidation, resulting in MDA accumulation [43]. Plants combat this through enzymatic and non-enzymatic antioxidant systems, including phenolics, flavonoids, and anthocyanins. Antioxidant enzymes such as POD, CAT, SOD, and APX are critical for detoxifying ROS [44]. SOD catalyzes the dismutation of superoxide into oxygen and H_2_O_2_, while CAT and APX convert H_2_O_2_ into water and oxygen, preventing oxidative stress [45,46,47]. Our results show that POD activity increased significantly in inoculated plants under drought (Figure 5), suggesting enhanced antioxidant capacity via fungal symbiosis. This likely reflects the cascade of redox-regulating enzymes. The observed increase in APX activity further supports the involvement of the ascorbate–glutathione cycle in managing oxidative stress [48]. ROS not only disrupt biochemical processes but also reduce crop productivity and quality. Our study demonstrated increased cob biomass in inoculated plants under drought, highlighting the potential of fungal endophytes to support yield (Figure 6). In well-watered conditions, inoculation had minimal effect on cob traits, likely due to the absence of stress. Under drought, however, inoculation-maintained cob weight and length, though cob width was unaffected, indicating a targeted benefit in yield protection.

The beneficial effects of fungal endophytes are influenced by their strain origin and degree of stress adaptation. Endophytes isolated from extreme environments, such as Antarctic plants, often exhibit unique metabolic traits that can enhance host resilience under harsh conditions [16,17,40,49]. Inoculating crops with such extremophilic fungi has the potential to improve tolerance to drought and other climate-related stressors, thereby contributing to increased productivity in challenging environments [50].

## 4. Materials and Methods

### 4.1. Plant Material

Certified corn seeds (Tuniche 2710^®^, Tuniche, Rancagua, Chile) were used, sown on 15 November 2023, in a field located in Villa Alegre, Maule Region, Chile (35°38′28.3″ S 71°42′03.8″ W). The plants under water deficit (W−) were no longer watered as of 1 January 2024, and remained so until harvest on 26 March 2024. The plants were inoculated (E+) twice with 10 mL of a water suspension of 1 × 10^6^–1 × 10^7^ mL^−1^ of a 1:1 mixture of *Penicillium chrysogenum* and *P. brevicompactum* fungi in the base of each plant [17]. The first inoculation was on 10 January 2024, and the second on 25 January 2024. The well-watered plants (W+) were under full irrigation and received the same inoculations as the water-deficient plants. Plants were spaced 50 cm apart within each row, and rows were spaced approximately 1 m apart. Water-deficient plants were separated from well-watered plants by five rows. The uninoculated controls (E−) were treated with two applications of 10 mL of water per plant on the same dates as the fungal inoculations. Soil water levels were estimated using gravimetric water content [51]. Soil samples were analyzed monthly until the end of the assay. Plants were assessed in the last week of February 2025, 90 days after sowing. All physiological and biochemical assays were performed at the end of the 90-day treatment period in each of the 50 plants per treatment group.

### 4.2. UAVs and Spectral Images Analysis

The images of the study areas were acquired on 8 March 2024, using a Dji Matrice 300 RTK (SZ DJI Technology Co., Shenzhen, China) and a Micasense Rededge Multiespectral sensor (MicaSense Inc., Seattle, WA, USA). The RedEdge sensor captures five discrete spectral bands: blue (475 nm, 20 nm bandwidth), green (560 nm, 20 nm bandwidth), red (668 nm, 10 nm bandwidth), red edge (717 nm, 10 nm bandwidth), and near-infrared (NIR) (840 nm, 40 nm bandwidth). It has a field of view (FOV) of 47.2°, which provides adequate coverage for most UAV-based agricultural applications.

The images were acquired under ideal weather conditions, with low cloud cover and close to noon, to avoid shady areas. The flight height was 50 m above ground level. The images were registered in continuous mode at 2 s intervals and a speed of 3.5 m s^−1^, resulting in side and forward overlaps equal to 90% and 80%, respectively, and in a 2.39 cm pixel size. On the other hand, it was not necessary to use Ground Control Points (GCPs) due to the inclusion of GNSS-IMU technology within the sensor. We used Agisoft Metashape software (Agisoft LLC; St. Petersburg, Russia; version 1.7.3) for photogrammetric processing. To obtain spectral metrics, vegetation indices (VIs) were computed using the spectral data provided by the RedEdge sensor. The VIs are spectral transformations that allow the analysis of vegetation properties and facilitate spatial comparisons of photosynthetic activity. The Vis are shown in Table 4.

Then, the 95th percentile, representing the threshold below which 95% of the data values lie and capturing the lowest value of the top 5% of the data, was extracted for each subplot. This feature was derived using the Python (3.9.13) libraries NumPy (1.23.0) (https://numpy.org/; accessed on 9 March 2024) and Rasterstats (https://pythonhosted.org/rasterstats/#; accessed on 9 March 2024). The extracted data for each labeled subplot (defined during the plot segmentation process) were then exported as a comma-separated values (CSV) file.

### 4.3. Photochemical Performance Analysis; Chlorophylls and Carotenoids Content

The photochemical efficiency of Photosystem II (PSII) was assessed in each of the 50 plants per treatment by determining the Fv/Fm ratio, following the method outlined by Maxwell & Johnson (2000) [57]. Comparative analysis of PSII efficiency across treatments was performed according to the approach described by Balbontín et al. [18].

Chlorophyll (Chl) and carotenoids (Car) were extracted from 100 mg of leaf tissue using 80% acetone. Absorbance was measured at 663 nm (Chl a), 646 nm (Chl b), and 470 nm (Car), and pigment concentrations were calculated using the spectrophotometric method described by Lichtenthaler and Wellburn [58].

### 4.4. Free Proline and Membrane Damage Assays

Free proline content in aerial tissues was quantified in each of the 50 plants per treatment following the method of Bates et al. (1973) [59]. Briefly, 100 mg of shoot tissue was flash-frozen in liquid nitrogen, homogenized in 1.2 mL of 3% sulfosalicylic acid, and centrifuged at 16,000× *g* for 20 min at room temperature. One milliliter of the resulting supernatant was combined with 2 mL of ninhydrin reagent [2.5% ninhydrin prepared in a solution of glacial acetic acid, distilled water, and 85% orthophosphoric acid in a 6:3:1 ratio]. The mixture was incubated at 90 °C for 1 h, then cooled on ice. Subsequently, 2 mL of toluene was added to extract the chromophore. The toluene phase’s absorbance was determined at 525 nm using a Multiskan SkyHigh Microplate Spectrophotometer (Thermo Fisher Scientific, Waltham, MA, USA). Proline concentrations were determined in biological triplicate using a standard curve as described by Morales-Quintana et al. [17].

Lipid peroxidation in aerial tissues was evaluated by measuring malondialdehyde (MDA) content as an indicator of membrane damage, following the method of Barrera et al. [60], which is based on the quantification of MDA as a marker of oxidative stress [61].

### 4.5. Antioxidant Enzyme Activity Assays

Antioxidant enzyme activities—including superoxide dismutase (SOD), catalase (CAT), peroxidase (POD), and ascorbate peroxidase (APX)—were quantified in leaf tissue from each of the 50 plants per treatment group. Fresh samples (500 mg) were ground in liquid nitrogen and homogenized in 50 mmol L^−1^ phosphate buffer (pH 7.8) containing 1% polyvinylpyrrolidone (PVP). The homogenate was centrifuged at 10,000× *g* for 20 min at 4 °C, and the resulting supernatant was used for enzyme activity assays. Enzyme activities were measured following the protocols outlined by Erdogan et al. [62]. All assays were performed in triplicate using independent biological replicates.

### 4.6. Cob Size and Weight Analysis

All cobs produced by inoculated (E+) and non-inoculated (E−) plants under both water-deprived (W−) and well-watered (W+) conditions were harvested (50 plants each treatment). Cob size, including length and middle width, was measured, and the weight of each cob was recorded.

### 4.7. Antioxidant Compound Assays

The antioxidant compounds were assessed in each of the 50 plants per treatment group. Total phenolic and flavonoid contents were quantified in leaf tissues. Samples were homogenized in 1% HCl in methanol (5 mL per gram of tissue) using a mortar and pestle. The extracts were stirred for 1.5 h at room temperature, centrifuged at 4200× *g*, and the supernatant was collected. For each sample, three independent extractions were performed from 10 g of tissue. The Folin–Ciocalteu method was assessed to determine the total phenolic content [63]. The absorbance was measured at 700 nm, and results expressed as grams of gallic acid equivalents per kilogram of fruit (g kg^−1^ GAE).

By the aluminum chloride colorimetric method, the total flavonoid content was assessed [64]. Absorbance was recorded at 415 nm, and results were expressed as grams of quercetin equivalents per kilogram of fruit (g kg^−1^ quercetin). All measurements were conducted in triplicate with both biological and technical replicates.

Anthocyanin concentration was determined using the pH differential method as described by Lee et al. (2008) [65] and standardized by Gil i Cortiella et al. [66]. Briefly, 0.15 mL of extract was mixed separately with 0.75 mL of potassium chloride solution (0.025 M, pH 1.0) and sodium acetate solution (0.4 M, pH 4.5). After incubation at room temperature for 50 min, absorbance was measured at 524 nm and 700 nm. Anthocyanin content was expressed as grams of cyanidin 3-glucoside equivalents per kilogram of fruit (g kg^−1^ Cy3G), using the formula A = (A_524_ − A_700_)_ph_ 1.0 − (A_524_ − A_700_)_ph_ 4.5, with a molar extinction coefficient of 26,900. Each analysis was conducted in triplicate with both biological and technical replicates.

### 4.8. Statistical Analysis

A two-way ANOVA was conducted to evaluate the effects of water availability (W) and fungal inoculation (E) on multiple physiological and biochemical parameters, including gravimetric water content, Fv/Fm ratio, chlorophyll and carotenoid contents, malondialdehyde (MDA) levels, proline concentration, antioxidant enzyme activities, kernel weight and size, total phenolics, flavonoid and anthocyanin contents, as well as multispectral imaging data. Prior to the analysis, data were tested for normality and homogeneity of variance. Tukey’s HSD post hoc test was used to identify statistically significant differences between treatments (*p* < 0.05). All statistical analyses and visualizations were performed using GraphPad Prism 10 (GraphPad Software Inc., San Diego, CA, USA).

## 5. Conclusions

This study demonstrates that inoculation with Antarctic-isolated fungal endophytes significantly enhances maize resilience to drought stress. Under field conditions, inoculated plants exhibited improved physiological and biochemical performance, as evidenced by increased photochemical efficiency, elevated NDRE, NDVI, and SIPI spectral indices, and enhanced pigment profiles. These benefits were accompanied by reduced oxidative damage, as shown by lower MDA accumulation and modulated antioxidant enzyme activities—particularly increased POD and APX—suggesting that endophyte-mediated stress mitigation involves both enzymatic and non-enzymatic antioxidant responses. Proline accumulation and phenolic compound dynamics further support the role of fungal symbiosis in maintaining cellular homeostasis under water-limited conditions. Notably, UAV-based spectral imaging proved effective in non-destructively detecting these physiological differences, offering a scalable tool for monitoring stress responses. Importantly, while fungal inoculation had a limited impact under well-watered conditions, it significantly improved cob biomass, length, and physiological status under drought, underscoring its potential for increasing yield stability in climate-challenged environments. Importantly, *Penicillium* species—used in this study—are globally widespread and are not classified as dangerous or high-risk organisms. However, the regulations vary significantly across countries, and the introduction of microorganisms isolated from foreign environments into outdoor agricultural systems is often subject to stringent legal restrictions. As a result, replicating the methodology used in this study may be challenging in certain regions due to biosafety and ecological protection policies. Overall, these findings highlight the promise of leveraging extremophile fungal endophytes and UAV technologies to bolster crop performance under water scarcity—a critical strategy for future sustainable agriculture.

## Figures and Tables

**Table 1:** Percentage of field capacity.

	% FC
W−	59.9 ± 4.0 ^b^
W+	100.0 ± 7.8 ^a^

Field capacity (FC) of soil treated (W+ = well-watered, W− = water-deficient). Different letters indicate significant differences (*p* < 0.05).

**Table 2:** Photochemical efficiency of photosystem II (Fv/Fm).

	E−	E+
W−	0.596 ± 0.167 ^c^	0.775 ± 0.023 ^b^
W+	0.794 ± 0.014 ^a^	0.798 ± 0.009 ^a^

Water availability (W+ = well-watered, W− = water-deficient) and inoculation (E− = non-inoculated, E+ = inoculated). Different letters indicate significant differences (*p* < 0.05).

**Table 3:** Chlorophyll and carotenoid content in leaves.

	W+E+	W+E−	W−E+	W−E−
Chlorophyll *a*	32.0444 ± 0.0266 ^a^	28.8428 ± 0.0299 ^b^	25.2858 ± 0.0090 ^c^	22.3437 ± 0.0257 ^d^
Chlorophyll *b*	25.6377 ± 0.0293 ^b^	13.4112 ± 0.0268 ^c^	32.1457 ± 0.0075 ^a^	8.5753 ± 0.0278 ^d^
Carotenoids	41.1279 ± 0.0255 ^b^	26.4645 ± 0.0249 ^c^	45.5467 ± 0.0119 ^a^	18.4337 ± 0.0229 ^d^

Water availability (W+ = well-watered, W− = water-deficient) and inoculation (E− = non-inoculated, E+ = inoculated). Different letters indicate significant differences (*p* < 0.05).

**Table 4:** Vegetation indices (VIs).

Vegetation Indices (VI)	Formula	Description	Reference
Normalized Difference Vegetation Index (NDVI)	(NIR − Red)/(NIR + Red)	It measures vegetation health by comparing the difference between near-infrared (NIR) and red light reflectance. NDVI is a reliable indicator of plant vigor and biomass.	[52]
Green Chlorophyll Index (GCI)	(NIR/Green) − 1	Index that focuses on estimating chlorophyll content in plant leaves, by comparing reflectance in the near-infrared (NIR) and green bands. GCI provides insights into the chlorophyll concentration, which is directly related to photosynthetic capacity and plant health.	[53]
Structure Insensitive Pigment Index (SIPI)	(NIR − Blue)/(NIR − Red)	Measures the ratio of pigments in vegetation, particularly focusing on the ratio of carotenoids to chlorophyll. This index is designed to be less affected by structural variations in the plant canopy, making it useful for assessing plant stress levels and detecting changes in pigment composition related to stress factors.	[54]
Green Normalized Difference Vegetation Index (GNDVI)	(NIR − Green)/(NIR + Green)	Emphasizes the presence of chlorophyll by using the green band instead of red, making it more sensitive to chlorophyll levels in the plant canopy.	[55]
Normalized Difference Red Edge Index (NDRE)	(NIR − Rededge)/(NIR + Rededge)	Designed to monitor vegetation health and stress by using the red edge band, which is sensitive to changes in chlorophyll content that occur before visible stress symptoms.	[56]

## Data Availability

The raw data supporting the conclusions of this article will be made available by the authors upon request.
